# Supplementary figures and images for: Chimeric crRNA improves CRISPR–Cas12a specificity in the N501Y mutation detection of Alpha, Beta, Gamma, and Mu variants of SARS-CoV-2
Source: PLoS One. 2021 Dec 23;16(12):e0261778. doi: 10.1371/journal.pone.0261778 (PMC8700041; doi:10.1371/journal.pone.0261778)

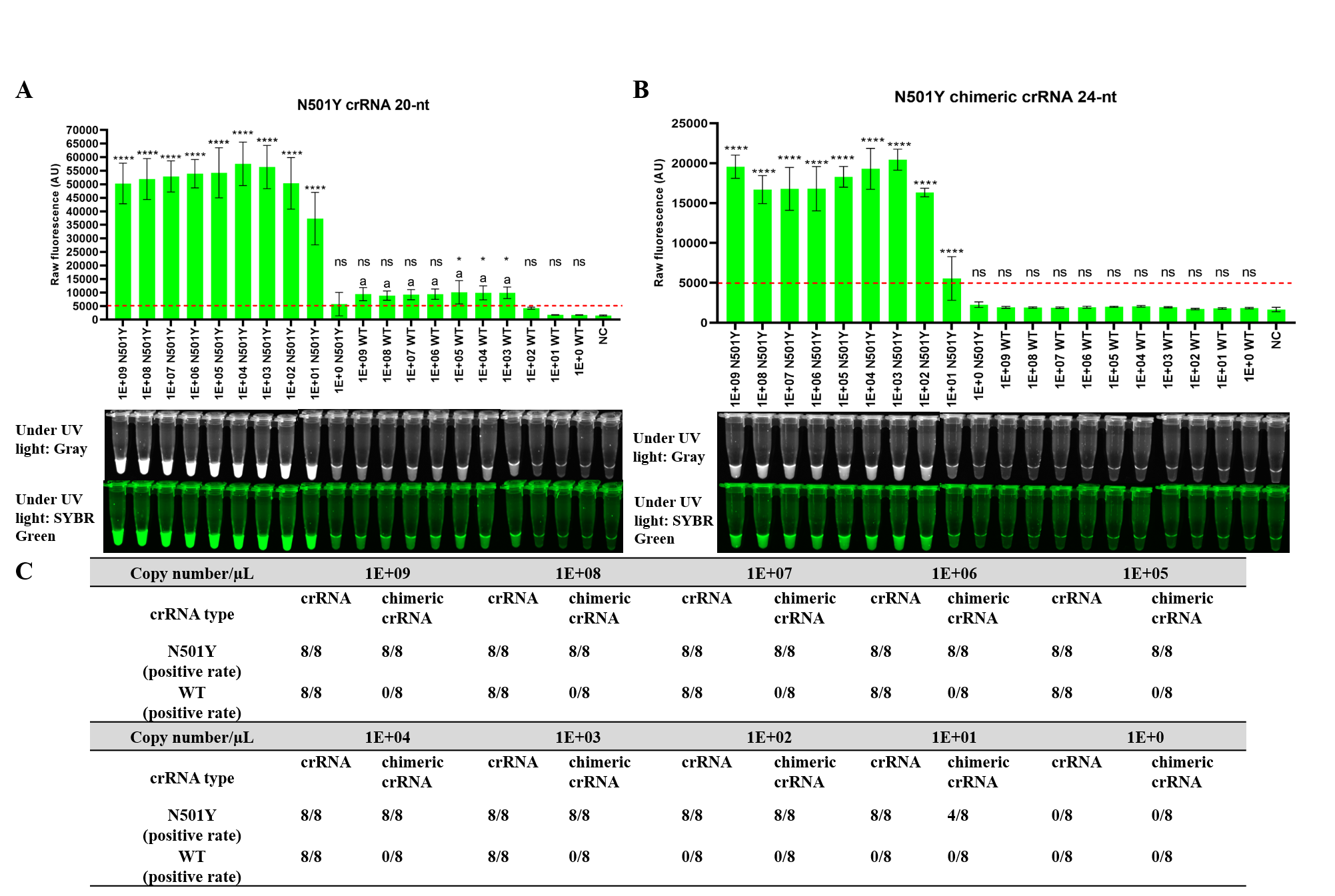

Supplement: S1 Fig — (A) Detection of N501Y variant and wild type using N501Y crRNA 20-nt after incubation for 1 h. The (a) indicates fluorescence of the wild type could be visualized. (B) Detection of N501Y variant and wild type using N501Y chimeric crRNA 24-nt after incubation for 1 h. No false positive signal is obtained. The horizontal red dashed lines indicate the threshold fluorescence signal that can be visualized under UV light. The threshold level of 5000 AU has been determined for visualizing under UV light and validated against spectrophotometry reading. NC stands for non-template control. Error bars represent the standard deviation of means. (C) Table summarizing positive rate of N501Y and wild type when using different crRNAs after incubation for 1 h. Eight replicates were run (n = 8). For (A) and (B), statistical analysis was performed using a one-way ANOVA test with Dunnett’s multiple comparisons test. Where the raw fluorescence (AU) of each reaction was compared to the respective NCs. The asterisks (*, **, ***, ****) indicate significant differences with p < 0.05, p < 0.01, p < 0.001, and p < 0.0001 and ns denotes not significant (p > 0.05). (TIF) [file pone.0261778.s001.tif]

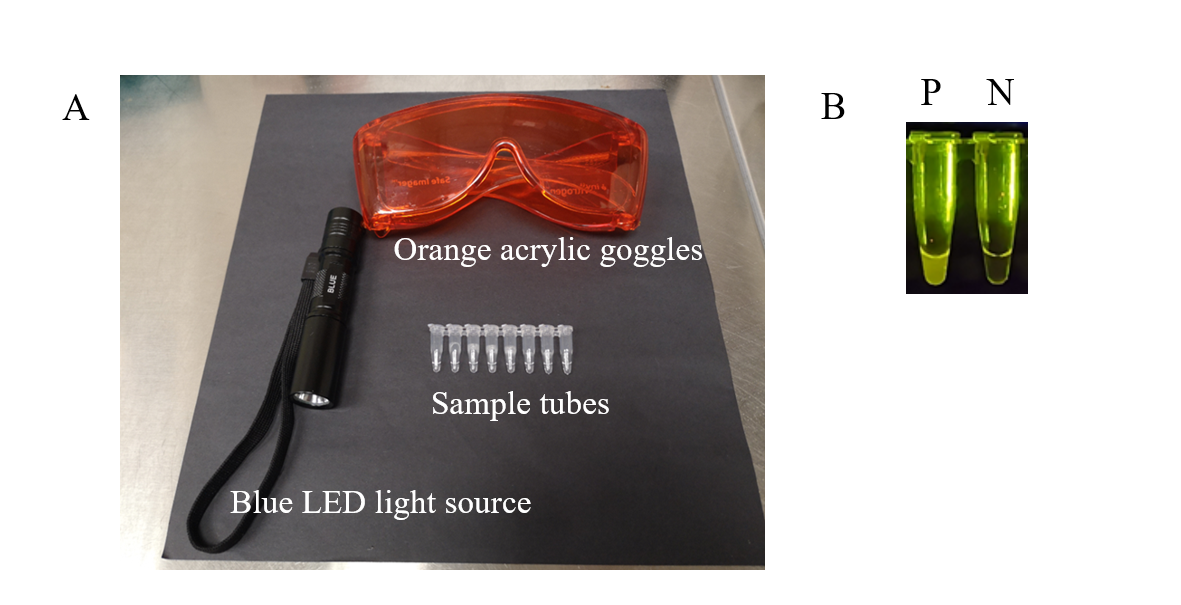

Supplement: S2 Fig — (A) Equipment used for signal visualization, including blue LED light, filter (an orange acrylic goggles (Invitrogen Safe Imager)), sample tubes, and black paper with a non-reflective surface. (B) Photograph of P (positive) and N (negative) samples under blue LED light. (TIF) [file pone.0261778.s002.tif]
