# Supplementary material for: Chimeric crRNA improves CRISPR–Cas12a specificity in the N501Y mutation detection of Alpha, Beta, Gamma, and Mu variants of SARS-CoV-2
Source: PLoS One. 2021 Dec 23;16(12):e0261778. doi: 10.1371/journal.pone.0261778 (PMC8700041; doi:10.1371/journal.pone.0261778)
Supplement: Attachment — Submitted filename: Comment.docx [file pone.0261778.s003.docx]

**Overall comments & recommendations**:

The aim of the study is the first report that chimeric crRNA could be useful for enhancing detection of the specificity of CRISPR-Cas12a for SARS-CoV-2 mutation N501Y, which is shared by Alpha, Beta, Gamma, and Mu variants of SARS-CoV-2 without reducing its sensitivity.

To test specificity, the authors developed N501Y chimeric crRNA 24-nt sequence and compared it with crRNA 20-nt to test N501Y variant and wild type of the virus. In addition, to determine whether the sensitivity of N501Y chimeric crRNA 24-nt is significant for N501Y detection, the limit of detection (LoD) was evaluated and compared to N501Y crRNA 20-nt using the tenfold serial dilution of synthetic RNA containing gene fragments of SARS-CoV-2.

The study provided valuable data on this chimeric crRNA. However, there are some comments on the study.

1. In the background section, the authors should explain how application of chimeric crRNA on N501Y detection is significant. And, the authors should further describe how to identify the variants of Alpha, Beta, Gamma, and Mu.

2. Please describe Statistical analysis in Method.

3. In Discussion, the effectiveness and mechanism of chimeric crRNA on N501Y is needed to discuss.
4. Lines149: Please describe the SARS-CoV-2 origins in the experiment
