# Supplementary material for: Chimeric crRNA improves CRISPR–Cas12a specificity in the N501Y mutation detection of Alpha, Beta, Gamma, and Mu variants of SARS-CoV-2
Source: PLoS One. 2021 Dec 23;16(12):e0261778. doi: 10.1371/journal.pone.0261778 (PMC8700041; doi:10.1371/journal.pone.0261778)
Supplement: Attachment — Submitted filename: Response to Reviewers.docx [file pone.0261778.s004.docx]

Review Comments to the Author

Please use the space provided to explain your answers to the questions above. You may also include additional comments for the author, including concerns about dual publication, research ethics, or publication ethics. (Please upload your review as an attachment if it exceeds 20,000 characters)

Reviewer #1: The baseline of the conclusion which chimeric crRNA improves the specificity of Cas12a-based detection of COVID-19 N501Y variant is acceptable and can be useful for the scientific community once the data is published. The following comments need to be addressed in the revision:

1. How does the LOD of 5,000 raw fluorescent units be selected? It seems that this is chosen in this paper with the intention to differentiate the detection result with crRNA 20-nt and chimeric crRNA 24-nt as false positive and negative, which makes the result more interesting. The more rigorous way to report specificity improvement using the fold change in background signal (e.g. ~ 3-fold) . Also, since the maximum signal of detection in chimeric crRNA also reduces, the author should compare the dynamic range of detection using different crRNA.

**Response:** Many thanks for your comments. It is very important issue you raised here. We have explained in the manuscript how 5000 AU was chosen as the threshold. For your kind information, below 5000 AU from the spectrophotometry reading, these test tubes are not visible under UV light. The signal of the N501Y and wild type of chimeric crRNA 24-nt is lower than that of crRNA 20-nt, so the fold change cannot give the exact picture of specificity in this case. That is why we have provided the data with 5000 AU as the threshold. We did not mention any dynamic range because when the signal is below 5000 AU, it cannot be visualized whereas at any value above 5000 AU, it has been visualized.

2. Statistical analysis of significance is not performed in all bar graphs where such analysis is needed and the method of statistical analysis is missing in the method section.

**Response:** Thank you very much for your suggestions. Statistical analysis results have been added to all bar graphs. We also added the method of statistical analysis in the method section. Please check line 198 of the revised manuscript with track changes.

Reviewer #2: Overall comments & recommendations:
The aim of the study is the first report that chimeric crRNA could be useful for enhancing detection of the specificity of CRISPR-Cas12a for SARS-CoV-2 mutation N501Y, which is shared by Alpha, Beta, Gamma, and Mu variants of SARS-CoV-2 without reducing its sensitivity.
To test specificity, the authors developed N501Y chimeric crRNA 24-nt sequence and compared it with crRNA 20-nt to test N501Y variant and wild type of the virus. In addition, to determine whether the sensitivity of N501Y chimeric crRNA 24-nt is significant for N501Y detection, the limit of detection (LoD) was evaluated and compared to N501Y crRNA 20-nt using the tenfold serial dilution of synthetic RNA containing gene fragments of SARS-CoV-2.
The study provided valuable data on this chimeric crRNA. However, there are some comments on the study.

1.In the background section, the authors should explain how application of chimeric crRNA on N501Y detection is significant. And, the authors should further describe how to identify the variants of Alpha, Beta, Gamma, and Mu.

**Response:** Many thanks for your comments. As we are the first to use chimeric crRNA on detection in order to find a method that can improve the specificity of CRISPR-Cas12a detection, thus, we have added how the application of chimeric crRNA is significant in the discussion section. For your kind information: the sensitivity of chimeric crRNA is comparable to regular crRNA, whereas the specificity is higher and can be stable for more than 2 h. It improves and provides the versatility of applying CRISPR-Cas12a in a larger throughput which will take more time to prepare reactions, even as a point-of-care testing (POCT). Please refer to line 147 of the revised manuscript with track changes

How to identify the variants of Alpha, Beta, Gamma, and Mu has also been added in the discussion. It is worthy to mention that, N501Y mutation is shared by Alpha, Beta, Gamma, and Mu; identification of N501Y is the first step of differentiating variants of Alpha, Beta, Gamma, and Mu from wild type and other variants. To further differentiate Alpha, Beta, Gamma, and Mu, other mutation sites in the Spike protein like 69-70 deletion or 144 deletion, 242-244 deletion or K417N, R190S or K417T, T95I or R346K can be detected respectively. Please refer to line 158 of the revised manuscript with track changes

2. Please describe Statistical analysis in Method.

**Response:** Thank you very much for your valuable comments. We added the method of statistical analysis in the method section. Please check line 198 of the revised manuscript with track changes

3.In Discussion, the effectiveness and mechanism of chimeric crRNA on N501Y is needed to discuss.

**Response:** Many thanks for your comments. The effectiveness and mechanism of chimeric crRNA on N501Y have been added in the discussion of the revised manuscript. For your kind information: The sensitivity of chimeric crRNA is comparable to regular crRNA, whereas the specificity is higher and it is stable for more than 2 h. Replacing the last 8-nt of the crRNA with DNA can decrease the binding energy between crRNA and target DNA, leading to less off-target of CRISPR-Cas12a [9]. RNA-guided Cas12a unleashes indiscriminate single-stranded DNase activity when CRISPR-Cas12a recognizes its target [11]. As the detection signal is produced by cleavage of fluorophore-quenched ssDNA fluorescent reporter, improving on-target specificity by chimeric crRNA 24-nt can increase the specificity of CRISPR-Cas12a detection. Please refer to line 147 and line 150 of the revised manuscript with track changes.

4.Lines149: Please describe the SARS-CoV-2 origins in the experiment

**Response:** We have mentioned the SARS-CoV-2 origins in the revised manuscript (line 165). The above mentioned SARS-CoV-2 gene fragments was collected form BGI (Beijing Genomics Institute, a Chinese genome sequencing company, headquartered in Shenzhen, Guangdong, China).
