# Supplementary material for: Chimeric crRNA improves CRISPR–Cas12a specificity in the N501Y mutation detection of Alpha, Beta, Gamma, and Mu variants of SARS-CoV-2
Source: PLoS One. 2021 Dec 23;16(12):e0261778. doi: 10.1371/journal.pone.0261778 (PMC8700041; doi:10.1371/journal.pone.0261778)
Supplement: Attachment — Submitted filename: Response to Reviewers.docx [file pone.0261778.s005.docx]

Reviewers' comments:

Reviewer's Responses to Questions

6. Review Comments to the Author

Please use the space provided to explain your answers to the questions above. You may also include additional comments for the author, including concerns about dual publication, research ethics, or publication ethics. (Please upload your review as an attachment if it exceeds 20,000 characters)

Reviewer #1: Minor suggestion: Figure 1C and 1D and the new Figure 2A and 2C look very similar, and my understanding is that they show results from the same experiments but at different time points after reaction incubation. To clarify the difference between Figure 1 and Figure 2 results, I recommend adding the time-point information directly on the figures.

**Response**: Many thanks for your suggestions. Time-point information has been added to Figure 1C and 1D and the new Figure 2A and 2C. Please check the revised Figure 1 and Figure 2. Time-point information has also been added to the legends of Figure 1 and S1 Figure. Please check lines 82 and 267 of the revised manuscript.
